# The prevalence of and exact indications for daily opioid use among aged home care clients with and without dementia

**DOI:** 10.1007/s40520-020-01627-8

**Published:** 2020-06-29

**Authors:** Heidi Mörttinen-Vallius, Sirpa Hartikainen, Lauri Seinelä, Esa Jämsen

**Affiliations:** 1 Faculty of Medicine and Health Technology, Tampere University, 33014 Tampere, Finland; 2 Research Centre of Geriatric Care, School of Pharmacy, University of Eastern Finland, Kuopio, Finland; 3 Faculty of Medicine and Health Technology and Gerontology Research Centre GEREC, Tampere University, 33014 Tampere, Finland; 4 Tampere University Hospital, P. O. Box 2000, 33521 Tampere, Finland

**Keywords:** Aged, Dementia, Home care, Opioids, Resident assessment instrument

## Abstract

**Background:**

The increasing trend of opioid use for non-malignant pain among older people has raised concerns about whether opioids are used for appropriate indications. On the other hand, pain in patients with dementia may be undertreated.

**Aims:**

To examine the prevalence of and indications for daily opioid use among home care clients, and to determine opioid use differs between those with and without dementia.

**Methods:**

All home care clients aged ≥ 65 years using opioids daily (*n* = 282) were identified based on their first Resident Assessment Instrument–Home Care assessment in 2014. Exact indications for opioid use, the opioid substance used, the median duration of use, and changes in opioid medication within 12 months from study entry were obtained from the electronic medical records.

**Results:**

The prevalence of daily opioid use was 9.3%, and the median duration of use before the study entry was 357 days (interquartile range 126–719 days). The majority of clients continued to use opioids daily during the follow-up year. Vertebral osteoporotic fractures (21.6%), degenerative spinal disorders (20.9%), and osteoarthritis (20.6%) were the most common indications for opioid use. Buprenorphine was used more frequently in persons with dementia, but otherwise there were no differences between those with and without dementia.

**Discussion and conclusions:**

Home care clients use opioids for long periods of time for pain related mostly to musculoskeletal disorders, although the effectiveness of long-term opioid use is not clear. The lack of effective or suitable options for management of pain might explain the situation.

## Introduction

The overall trend of opioid use for non-malignant pain has been increasing among older people regardless of the living setting [1–3]. Opioid users are more often women, aged (≥ 80 years), and from a lower socioeconomic position. They tend to more often have cardiovascular diseases, diabetes, cancer, rheumatoid arthritis, hip fractures, and osteoporosis compared to nonusers [4, 5].

Daily pain associates with many of the same characteristics and diseases as opioid use: female gender, osteoarthritis, osteoporosis, rheumatoid arthritis, history of fractures, ischemic heart disease, peripheral vascular disease, depressive symptoms, frailty [6–9]. The most common sites of pain are the lower back and lower limbs, specially the joints [10]. The reported prevalence of daily pain has been reported to be up to 60% of the study population among older home care clients in previous studies [7, 9].

Patients with cognitive impairment have been found to be at risk for undetected or undertreated pain [6, 7, 11], even though the prevalence of pain-related diseases has been the same [12, 13]. Self-report has been recommended as the gold standard for detecting pain [14]. However, patients with dementia have a reduced ability to verbalize and remember pain experiences, which exposes them to the undertreatment of pain [15]. Earlier findings indicate that the frequency of opioid use is lower among older people with dementia in community-dwelling [5, 16], home care [7, 17], and institutionalized settings [4, 13, 18]. However, some studies have shown an equal or higher frequency of opioid use among older people with dementia [4, 12, 19].

The increasing trend of opioid use has raised concerns about whether opioids are used for appropriate indications [20]. The aim of this study was therefore to examine the prevalence of and the indications for regular opioid use among older home care clients, and to investigate differences in opioid use between those with and without dementia.

## Methods

This study was based on data from the Resident Assessment Instrument–Home Care (RAI–HC) that was supplemented by a retrospective review of local medical records. The study population consisted of persons aged ≥ 65 years receiving regular home care services at least once per week in the area of Tampere city (population circa 222,000, of which 17.9% are aged ≥ 65 years), Finland during 2014. In this area, approximately 9% of inhabitants aged ≥ 65 years received home care services in 2014. All citizens in Finland have access to public tax-financed health care services, and the majority of home care clients in the area were treated either by a geriatrician or general practitioner in 2014.

The RAI-HC has been part of home care services in Tampere since 2007. Clients are evaluated by educated nursing staff approximately every six months or when a notable change in the state of health occurs. The RAI-HC is an international and widely used instrument designed for the comprehensive, multidimensional assessment of older people living with disabilities or receiving supportive services in community-based settings (www.interrai.org). It contains clients’ socio-demographic variables, clinical diagnoses, medications, and the physical, psychological, cognitive, and social status, and it features several standardized sum scales to measure clients’ disabilities and state of health. The reliability and validity of the instrument have been reported previously elsewhere [21, 22].

The Pain scale [23] in the RAI-HC assesses pain over the seven days before the assessment and is based on items on pain frequency and pain intensity. It ranges from 0–3, where scores ≥ 2 refer to daily pain. The intensity of pain is scored from mild to severe. The Activities of Daily Living hierarchy scale (ADLH) [24] is based on items on eating, locomotion, personal hygiene, and toilet transfer ranging from independent in all four (ADLH = 0) to total dependence in all four (ADLH = 6). The CHESS (Changes in Health, End-Stage Disease, Signs and Symptoms Scale) [25] represents the level of instability in health, and scores ≥ 3 describe moderate to very high health instability. The Cognitive Performance Scale (CPS) has been validated against the Mini-Mental State Examination [26]. Scores range from 0–6, with higher scores indicating more severe impairment. Depression Rating Scale (DRS) [27] (range 0–14) scores ≥ 3 indicate possible depression. Other variables, including the need for a walking aid, living alone, body mass index, and behavioural symptoms (one or more of the following: wandering, verbal or physical aggression, oppositional or socially inappropriate behaviour) were also derived from the RAI-HC. For this study, the data were gathered from the client’s first RAI-HC assessment during 2014.

**Fig. 1:**
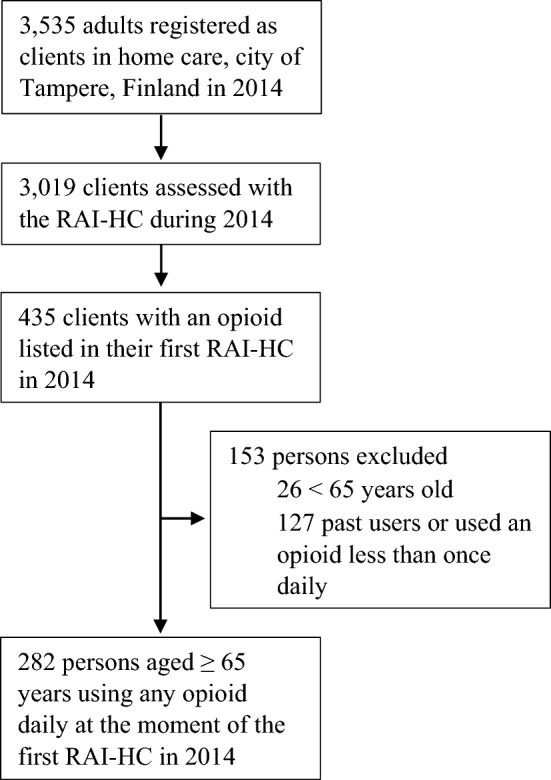
Population selection

Home care clients using opioids daily, based on the medications listed in each client’s first assessment with the RAI-HC during 2014, were included in this study. Opioid use was checked manually by one author (H.M.V.) against the electronic medical records. The records cover both home care and primary care health centres and Tampere’s municipal secondary care hospital. Two groups were excluded: clients noted to be past users (persons in whom earlier opioid use had been stopped before the RAI assessment), and clients who used opioid less frequently than once daily at the time of the first RAI-HC as it was not possible to verify retrospectively opioid doses these clients used, or if used at all. The population selection is described in Fig. 1.

The specific opioid substance as well as the starting date and changes in opioid medication during the next 12 months were recorded retrospectively for each opioid user starting from the date of the first RAI-HC measurement in 2014. The duration of daily opioid use before the first RAI-HC in 2014 was checked from the local electronic medical records (available since 2001). Maximum interruptions of seven days in the use of the same opioid and changes from one opioid to another without interruptions were accepted for this period of long-term use before study entry. The oral morphine equivalent daily dose (MEDD) [28] was calculated for each opioid user at the moment of the study entry. The opioids on the market in Finland at the time were fentanyl, morphine, hydromorphone, oxycodone, buprenorphine, tramadol, and codeine. In this study population, codeine was used only in combination with paracetamol.

Indications for opioid use and dementia diagnosis were noted from the medical records. An indication or concurrent indications for current opioid use were recorded according to the ICD-10 classification from the documentation of the prescribing physician. In the case of missing ICD-10 codes, indications were categorized by a study physician based on the description of the disease, symptoms, and status in the medical records. The categorization of indications for opioid use is shown in Table 1. The indication for opioid use was defined as unknown when it was not possible to specify the reason for the prescription.

**Table 1:** Categorization of recorded indications for opioid use

Musculoskeletal disorders	
Vertebral fractures (osteoporotic)	
Degenerative spine disorders	Intervertebral disc and facet join degeneration Spondylosis Spondylolisthesis Scoliosis Unspecified low back pain The ICD-10 code of spinal stenosis caused by degenerative lesions of spine when used without a neuropathic component of pain
Osteoarthritis	
Other fractures and injuries	
Muscular pain or tendinopathy	Rotator cuff syndrome Greater trochanteric pain syndrome Unspecified myalgia
Arthritis	Rheumatoid arthritis Gout Unspecified arthritis
Other conditions	
Neuropathic pain	Polyneuropathy Radiculopathy caused by intervertebral disc and facet joint degeneration Spinal stenosis Postherpetic neuralgia Carpal tunnel syndrome Phantom limb pain
Cardiovascular diseases	Chronic venous insufficiency Venous leg ulcers Lower peripheral arterial disease Ischaemic ulcers Cardiac oedema Coronary artery disease Ulcers caused by cutaneous vasculitis
Surgery	
Cancer	
Psychiatric conditions	Persistent somatoform pain disorder Prescription drug dependence
Other neurologic diseases	Spasticity (cerebral infarction) Rigidity (Parkinson disease) Restless legs syndrome
Gastrointestinal symptoms	Unspecified gastrointestinal pain
Decubitus ulcer	

Specific diagnoses of dementia were set by a geriatrician or a neurologist. The diagnostic process included the evaluation of a patient’s cognitive impairment, neuropsychological tests, brain imaging, and a clinical examination, as per the national guidelines and the ICD-10 criteria of dementia. In this study, a person was defined as having dementia when the complete diagnostic process documented in the medical records was concluded by the moment of the first RAI-HC in 2014, independent of the exact diagnosis of memory disorder.

Data on comorbidities that could affect opioid use [4, 5] and could enable a comparison with other populations and studies were gathered from the medical records. These included any documented current cancer (except basalioma), depression or anxiety disorder, ischemic heart disease, hypertension, atrial fibrillation, congestive heart failure, history of cerebral infarction, diabetes, chronic pulmonary disease (asthma, chronic obstructive pulmonary disease, or pulmonary fibrosis), rheumatoid arthritis, and osteoarthritis (radiologically confirmed).

The study protocol was approved by the city of Tampere. Persons in this study were not contacted, and as the retrospective study protocol did not have an effect on their treatment, neither ethics board approval nor informed patient consent was required by Finnish law.

### Statistical analysis

Data were analysed using SPSS version 25. *p* values < 0.05 were considered statistically significant. Descriptive analyses were completed using percentages, means with standard deviations (SD), or medians with interquartile range (IQR). Comparisons between opioid users with and without dementia were made using the independent samples *t* test for continuous variables (except the duration of constant opioid use before the first RAI-HC and MEDDs where the Mann–Whitney *U* test was used) and cross-tabulation with the Chi-square test or Fisher’s exact test for nominal variables. All the results are reported as two-tailed. There were no missing values except body mass index, which was not recorded for 24 persons, and the RAI-HC measures, which were not recorded for 2 persons.

## Results

A total of 282 persons—9.3% of the home care clients assessed with the RAI-HC—were using opioids daily at the time of their first RAI-HC in 2014. The mean age of opioid users was 82.8 years (SD 7.3, range 65–99 years), and 79.4% were women (Table 2).

**Table 2:** Characteristics of all opioid users and those with and without dementia

Characteristics	All (*n* = 282)	With any dementia (*n* = 88)	Without dementia (*n* = 194)	*p* value^c^
Age, years [mean (SD)]	82.8 (7.3)	83.1 (6.4)	82.7 (7.7)	0.718
Gender, female (%)	79.4	78.4	79.9	0.775
Body mass index [mean (SD)]	26.8 (6.2)	25.8 (5.4)	27.3 (6.5)	0.064
Living alone (%)	80.9	78.4	82.0	0.483
Walking aid at home (%)	77.7	78.4	77.3	0.839
Performance in ADLs (ADLH)				0.313
Independent or need for supervision only (0–1) (%)	85.0	80.2	87.1	
Limited or extensive need for help (2–3) (%)	9.3	12.8	7.7	
Maximal need for help or fully dependent (4–6) (%)	5.7	7.0	5.2	
Unstable health state (CHESS^a^ ≥ 3) (%)	14.3	17.4	12.9	0.315
Cognitive performance scale (CPS)				< 0.001
Intact or borderline (0–1) (%)	62.9	22.1	80.9	
Mild impairment (2) (%)	31.4	62.8	17.5	
Moderate impairment (3–4) (%)	3.9	10.5	1.0	
Severe impairment (5–6) (%)	1.8	4.7	0.5	
Presence of behavioural symptoms^b^ (%)	10.6	18.2	7.2	0.006
Depression rating scale (DRS) ≥ 3 (%)	24.3	33.7	20.1	0.014
Chronic conditions				
Osteoarthritis (%)	80.1	80.7	79.9	0.878
Hypertension (%)	80.1	78.4	80.9	0.623
Atrial fibrillation (%)	33.7	37.5	32.0	0.362
Ischemic heart disease (%)	33.0	27.3	35.6	0.170
Congestive heart failure (%)	31.6	27.3	33.5	0.297
Diabetes (%)	26.2	22.7	27.8	0.366
Depression or anxiety disorder (%)	22.7	22.7	22.7	0.993
Chronic pulmonary disease (%)	18.4	18.2	18.6	0.940
Cerebral infarction (%)	17.7	26.1	13.9	0.013
Arthritis (%)	6.4	3.4	7.7	0.169
Cancer (%)	6.4	1.1	8.8	0.015
Pain scale				0.033
No (%)	7.5	7.0	7.7	
Less than daily (%)	18.9	25.6	16.0	
Daily, mild to moderate (%)	40.0	45.3	37.6	
Daily, severe (%)	33.6	22.1	38.7	
Morphine equivalent daily dose, mg [median (IQR)]	20.0 (11.0–30.0)	22.0 (11.0–22.5)	20.0 (11.0–30.0)	0.724

^a^Changes in Health, End-stage disease, Signs and Symptoms scale

^b^One or several of the following: wandering, verbal or physical aggression, oppositional or socially inappropriate behaviour

^c^Difference between those with and without dementia

Most of the opioid users were living alone, using a walking aid, and were either independent or needed at most supervision in eating, locomotion, personal hygiene, or toileting (Table 2). Dementia was diagnosed for 88 (31%) opioid users: 34 had Alzheimer’s disease, 13 vascular dementia, 30 both Alzheimer’s and vascular dementia, and 11 had other disorders (normal-pressure hydrocephalus, Parkinson’s disease dementia, dementia with Lewy bodies, or unknown dementia). The majority of them had at most mild cognitive impairment (Table 2). Of the opioid users without a dementia diagnosis, 19% (37 persons) had CPS scores ≥ 2, indicating at least mild cognitive impairment. The opioid users with dementia were more likely to have a history of cerebral infarction and less likely to have a current cancer diagnosis. Otherwise the prevalence of comorbid diseases was similar in opioid users with and without dementia. Based on the DRS, depressive symptoms were more common among the opioid users with dementia, but there was no difference in diagnoses of depression or anxiety disorder between persons with and without dementia.

Three-quarters (74%) of all opioid users had daily pain. Opioid users with dementia more frequently reported pain less than daily and less frequently reported severe daily pain compared to opioid users without dementia (Table 2).

Non-malignant diseases comprised the majority of indications for opioid use. Only 3.2% of the study population used an opioid for cancer-related pain (Table 3). Musculoskeletal disorders were the indication for opioid use for over four-fifths of the study population and the most common were vertebral osteoporotic fractures, degenerative spinal disorders, and osteoarthritis (21.6%, 20.9%, and 20.6%, respectively). Other acute fractures or fall-related injuries, muscular pain, and tendinopathy and arthritis were minor reasons within this group. Neuropathic pain was the indication for opioid use for 13.1% of the study population, and other rare indications were cardiovascular diseases, surgery, other neurologic diseases, psychiatric conditions, gastrointestinal symptoms, and decubitus ulcer. Fifty-four persons (19%) used opioids for more than one indication concomitantly. The reason for opioid prescription could not be traced from the medical records for 9.6% of the study population.

**Table 3:** Indications for opioid prescription of all opioid users and those with and without dementia

Indications for opioid prescription	All indications, % (*n* = 350)	All opioid users, % (*n* = 282)	Dementia status		
			With any dementia, % (*n* = 88)	Without dementia, % (*n* = 194)	*p* value^a^
Musculoskeletal disorders					
Vertebral fractures (osteoporotic)	17.7	21.6	23.9	20.6	0.540
Degenerative spine disorders	17.1	20.9	20.5	21.1	0.897
Osteoarthritis	16.6	20.6	22.7	19.6	0.546
Other fractures and injuries	8.3	10.3	12.5	9.3	0.409
Muscular pain or tendinopathy	5.4	6.7	8.0	6.2	0.583
Arthritis	2.3	2.8	1.1	3.6	0.442
Other conditions					
Neuropathic pain	10.9	13.1	12.5	13.4	0.835
Cardiovascular diseases	4.3	5.3	3.4	6.2	0.405
Surgery	3.1	3.9	3.4	4.1	1.000
Cancer	2.6	3.2	1.1	4.1	0.282
Other neurologic diseases	1.4	1.8	2.3	1.5	0.649
Psychiatric conditions	1.1	1.4	0.0	2.1	0.313
Gastrointestinal symptoms	0.6	0.7	1.1	0.5	0.527
Decubitus ulcer	0.6	0.7	1.1	0.5	0.527
Unknown	8.0	9.6	6.8	10.8	0.289

The cumulative percentage of all opioid users and opioid users with and without dementia is over 100.0%, because one person may use an opioid for more than one reason at the same time

^a^Difference between those with and without dementia

Weak opioids (codeine or tramadol) were used by 22.3% of the study population, buprenorphine by 61.7%, and strong opioids (fentanyl, morphine or oxycodone) by 18.1%. Six persons used concomitantly two opioids daily: one oxycodone and codeine combination and five buprenorphine and codeine combinations. Buprenorphine (all but one transdermal) was the most commonly used opioid (61.7%; median dose 10 μg/h, range 5-20 μg/h, one sublingual 1.6 mg per day), followed by codeine (combined with paracetamol; 15.2%; median daily dose 79 mg, range 30–180 mg), oxycodone (14.2%; median daily dose 17.5 mg, range 5–120 mg), tramadol (7.1%; median daily dose 100 mg, range 50–300 mg), transdermal fentanyl (2.1%; median dose 18.5 μg/h, range 12–75 μg/h), and morphine (1.8%; median daily dose 10 mg, range 8–20 mg). None of the study population used hydromorphone.

The median duration of opioid use before the study entry was 357 days (interquartile range 126–719 days, maximum 4,163 days i.e. approximately 11.4 years). Of all opioid users 31 (11.0%) died during the follow-up year. Approximately every sixth user (*n* = 50, 17.7%) stopped opioid use during the follow-up year or before death. Of the 232 persons (82.3%) who still used opioids at the end of their follow-up period (12 months or until death), 201 (71.3% of all opioid users) used an opioid daily during the whole follow-up period with no interruptions. During the follow-up period, 90 (31.9%) persons switched from one opioid to another: 67 persons used altogether two different, 18 persons three different, four persons four different, and one person five different opioids during the follow-up.

**Fig. 2:**
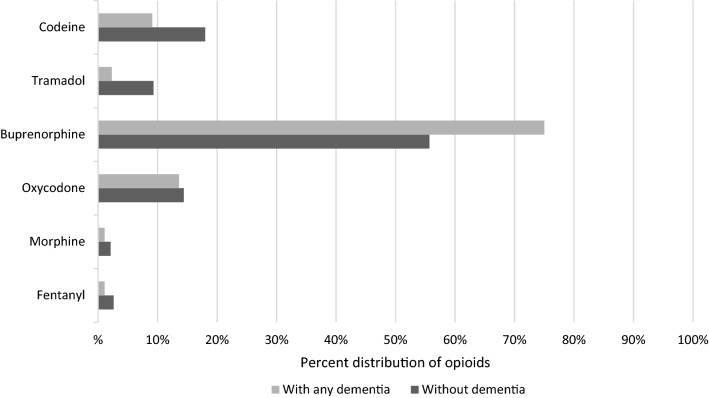
Proportion of home care clients using different opioids according to their dementia status. The cumulative percentage is over 100.0% because six clients (two with dementia and four without dementia) used more than one opioid daily at the same time

Among the opioid users with dementia, buprenorphine was more commonly used (75.0% vs. 55.7%, p = 0.002) and the proportions of weak opioids, tramadol (2.3% vs. 9.3%, *p* = 0.034) and codeine (9.1% vs. 18.0%, *p* = 0.053), were smaller than in the opioid users without dementia (Fig. 2). There were no differences in the indications for opioid use between those with and without dementia (Table 3), the median duration of daily opioid use before the study entry (376 days vs. 352 days, *p* = 0.383) or changes in opioid use during the follow-up period (stopping opioid use 22.7% vs. 15.5%, using an opioid daily 67.0% vs. 73.2%, having interruptions in opioid use 10.2% vs. 11.3%, in opioid users with and without dementia, respectively, *p* = 0.334). The median MEDDs used did not differ between home care clients with and without dementia (Table 2).

## Discussion

The prevalence of daily opioid use was about 9% in this population-based study of older home care clients. Musculoskeletal disorders—mostly vertebral osteoporotic fractures, degenerative spinal disorders, and osteoarthritis—were the indication for opioid use in over four-fifths of the home care clients, whereas the proportion of use for malignant pain was small. The reasons for and the pattern of opioid use did not differ between those with and without dementia, with the exception that the proportion of buprenorphine users was higher and weak opioids lower among those with dementia. Prolonged opioid use was common, and the majority of opioid users were still using an opioid daily at the end of the follow-up.

The prevalence of daily opioid use in the present study was quite consistent with the previous studies concerning community-dwelling older adults [5, 12, 29] [3, 16] in both Europe and the United States. However, it was significantly lower than among Medicare home health recipients in the United States [17]. The different definition of opioid use explains the more frequent use of opioids (27.5% of those with dementia and 16.9% of those without dementia among home-living persons aged ≥ 65 years) in a Danish study [4].

The main indications for daily opioid use were common conditions that have been associated previously with both opioid use and pain in older home care clients [7, 9, 30] and community-dwelling older adults [3–5]. The small proportion (3.2%) of use for malignant pain in this study is consistent with the previous finding that among aged cancer patients, only about half of the current pain is cancer-related [31] and only 6.4% of the opioid users had current cancer. However, this emphasizes the increasing trend of non-malignant indications for opioid use. Gastrointestinal symptoms, persistent somatoform pain disorder, and known prescription drug dependence as indications raised doubts regarding inappropriate prescribing practices, but the proportion was small. Of greater concern is that every tenth indication remained unknown despite a thorough search of the medical records. In many cases this was due to an inadequate examination of a patient and a description of symptoms, especially outside official appointments or home visits when a physician has answered to home care nurses’ consultations. If opioid use started during a hospital stay, the indication was often not recorded even though the reason for hospitalization did not explain opioid use. It was also common to renew opioid prescriptions without any evaluation of current pain.

The lack of effective or suitable options for pain treatment might explain the indications for opioid use in the present study. The previous systematic reviews have not found clinically important difference between paracetamol and placebo for pain, disability, or quality of life associated with acute lower back pain or osteoarthritis [32, 33]. Among older people, the use of non-steroidal anti-inflammatory drugs is often restricted only to the short term because of adverse effects on cardiovascular diseases, renal impairment, and the risk of gastrointestinal bleeding [14, 34]. Also, access to non-pharmacological therapies may be dependent on the availability of educated staff. The benefits of using opioids in the treatment of musculoskeletal disorders are, however, also unclear. The previous evidence from meta-analyses has not particularly supported the use of any opioid to treat chronic lower back pain or hip and knee osteoarthritis based on the modest or even questionable pain reduction compared to placebo [35, 36].

Although the most commonly used transdermal buprenorphine has several advantages—like ease of administration and unchanged metabolism in renal insufficiency [37]—which may explain its frequent use, its adverse effects are similar to other opioids. The evidence on the effectiveness of long-term opioid use for pain, physical function, and quality of life is also lacking [10, 35, 38]. These concerns are clinically important, as the present study indicates that home care clients use opioids, mostly buprenorphine, for very long periods of time. On the other hand, persistent pain is associated with poor self-rated health [39] and the risk of developing a disability in performing activities of daily living [9].

Except for the more frequent use of buprenorphine in persons with dementia, there was no association between dementia and how or why opioids were used among home care clients. The difference in the prevalence of cancer and previous cerebral infarction between clients with and without dementia did not impact the results due to the rarity of both diagnoses as an indication for opioid use. Contrary to the speculation presented in a previous Danish study [4], there were no signs of opioid use for the behavioural symptoms of dementia in this study. In long-term care, the situation might be different.

The study has certain limitations. Firstly, as only opioid users were included, the present study cannot answer the question whether home care clients with dementia used opioids more or less frequently than those without dementia. It is also unclear if there was a difference between persons with and without dementia who suffered pain and did not use opioids due to fear of adverse effects. However, the result does not suggest that pain is untreated among home care clients with dementia compared to those without dementia. It should be noted that most persons with dementia in this study had only mild cognitive impairment, and the results might be different in the population with more severe impairment.

Secondly, there was no available data on opioid purchases from pharmacies within this study. The appropriateness of the opioid prescriptions could not be assessed either. However, a study physician checked regular renewals of opioid prescriptions and the medication was checked by home care nurses for most of the home care clients. Furthermore, the original documentation in the medical records of the physician responsible for opioid use was used in this study from several years earlier. This made it possible to find out the exact indications and concurrent indications for opioid use, not only those diseases or other characteristics with which the opioid use was associated. The dementia diagnoses were also verified from the medical records. These facts give an advantage compared to the register-based studies or self-report. The regional representativeness of the population of interest was good, as only circa 15% of home care clients in the catchment area were not included due to the missing RAI-HC assessment.

Finally, due to the population selection method, it was not possible to find out the indications for all opioid initiations among home care clients in the present study. Persons using opioids only for a short period of time due to acute illness, especially after being admitted to a hospital, were probably missing. For this reason, the proportions of opioid use related to fractures and fall-related injuries, surgery, and cardiovascular diseases were probably smaller than they would have been if all opioid use had been studied for a fixed period of time. The practice of opioid treatment may vary between specialties and differ from the present results, for example in rural areas, due to different population characteristics.

## Conclusions

Home care clients use opioids for long periods of time for pain, mostly due to musculoskeletal disorders, though the effectiveness of long-term opioid use in these disorders has not been shown. Diagnosed dementia was not associated with how long or for what indications the opioids were used among aged home care clients.

## Data Availability

The Resident Assessment Instrument data that support the findings of this study were used under license for the current study, and so are not publicly available.
